# Clinical Impact of Tumor-Infiltrating Lymphocytes and PD-L1-Positive Cells as Prognostic and Predictive Biomarkers in Urological Malignancies and Retroperitoneal Sarcoma

**DOI:** 10.3390/cancers12113153

**Published:** 2020-10-27

**Authors:** Makito Miyake, Shunta Hori, Takuya Owari, Yuki Oda, Yoshihiro Tatsumi, Yasushi Nakai, Tomomi Fujii, Kiyohide Fujimoto

**Affiliations:** 1 Department of Urology, Nara Medical University, Nara 634-8522, Japan; horimaus@naramed-u.ac.jp (S.H.); tintherye@naramed-u.ac.jp (T.O.); K197610@naramed-u.ac.jp (Y.O.); takuro.birds.nest@gmail.com (Y.T.); nakaiyasusiuro@live.jp (Y.N.); kiyokun@naramed-u.ac.jp (K.F.); 2 Department of Diagnostic Pathology, Nara Medical University, Nara 634-8522, Japan; fujiit@naramed-u.ac.jp

**Keywords:** tumor-infiltrating lymphocyte, immune cells, tumor microenvironment, prognosis, immune checkpoint inhibitor, treatment response, urothelial carcinoma, renal cell carcinoma, prostate cancer, retroperitoneal sarcoma

## Abstract

**Simple Summary:**

Two host-dependent biological characteristics, “avoiding immune destruction” and “tumor-promoting inflammation” have been added to cancer hallmarks in 2011. The interaction and cross-talk among tumor cells and several immune cells in a tumor microenvironment are dynamic and complex processes. The purpose of this review is to discuss the prognostic impact of tumor-infiltrating lymphocytes and predictive biomarkers for immune checkpoint inhibitors in four urological solid tumors, the urothelial carcinoma, renal cell carcinoma, prostate cancer, and retroperitoneal sarcoma, through summarizing the findings of observation studies and clinical trials.

**Abstract:**

Over the past decade, an “immunotherapy tsunami”, more specifically that involving immune checkpoint inhibitors (ICIs), has overtaken the oncological field. The interaction and cross-talk among tumor cells and several immune cells in the tumor microenvironment are dynamic and complex processes. As immune contexture can vary widely across different types of primary tumors and tumor microenvironments, there is still a significant lack of clinically available definitive biomarkers to predict patient response to ICIs, especially in urogenital malignancies. An increasing body of evidence evaluating urological malignancies has proven that tumor-infiltrating lymphocytes (TILs) are a double-edged sword in cancer. There is an urgent need to shed light on the functional heterogeneity in the tumor-infiltrating immune system and to explore its prognostic impact following surgery and other treatments. Notably, we emphasized the difference in the immunological profile among urothelial carcinomas arising from different primary origins, the bladder, renal pelvis, and ureter. Significant differences in the density of FOXP3-positive TILs, CD204-positive tumor-infiltrating macrophages, PD-L1-positive cells, and colony-stimulating factors were observed. This review discusses two topics: (i) the prognostic impact of TILs and (ii) predictive biomarkers for ICIs, to shed light on lymphocyte migration in four solid tumors, the urothelial carcinoma, renal cell carcinoma, prostate cancer, and retroperitoneal sarcoma.

## 1. Introduction

The interaction and cross-talk among tumor cells and several immune cells in a tumor microenvironment are dynamic and complex processes [1,2]. In 2011, Hanahan and Weinberg defined “avoiding immune destruction” and “tumor-promoting inflammation” as emerging cancer hallmarks [3], which are host-dependent biological characteristics play crucial roles in the immune cell-mediated orchestration of tumor proliferation, progression, angiogenesis, epithelial-to-mesenchymal transition (EMT), invasion, and metastasis [4,5]. Although inflammation caused by the innate immune system was originally designed to fight infections and heal wounds, “tumor-promoting inflammation” can inadvertently contribute to multiple cancer hallmark capabilities by supplying active molecules to the tumor microenvironment [3,6,7,8]. “Avoiding immune destruction” allows tumor cells to escape immunosurveillance; the main role of T lymphocytes, B lymphocytes, macrophages, natural killer (NK) cells, neutrophils, and dendritic cells [3,9,10].

An increasing body of oncological research has provided evidence to suggest that tumor-infiltrating lymphocytes (TILs), including T lymphocytes such as CD8+ T cells and regulatory T cells, and NK cells such as tumor-associated NK cells and tumor-infiltrating NK cells, are double-edged swords in cancer [9,11,12]. The density, composition, and types of TILs vary greatly across tumor stage [10] and tumor entity [13]. Moreover, these features display significant heterogeneity between patients with the same type of tumors [14,15]. Lymphocyte migration to neoplastic lesions is mainly controlled by chemotactic factors, including chemokines and small cytokines, which are secreted from immune cells and tumor cells [16]. There are three types of immunological profiles: (1) immunologically “tumors” present with a high degree of T cell infiltration (e.g., melanoma, non-small-cell lung carcinoma, and renal cell carcinoma (RCC)) [17,18]; (2) immunologically “cold tumors” present with scarce immune infiltrates (e.g., prostate cancer (PCa) and pancreatic cancer) [19,20]; (3) immunologically “altered tumors” in which peri-tumoral sites are densely inflamed with immune cells which lack the capability to infiltrate the tumor [21]. Classification of tumors according to their immune phenotype can help predict responses to immune checkpoint inhibitors (ICIs), such as anti-programmed cell death 1 (PD-1) inhibitors, pembrolizumab, and nivolumab. Higher levels of immune cell infiltration and interferon signature (a T-cell-inflamed phenotype) are associated with a positive response to ICIs [19]. However, many other solid tumors fail to respond to ICIs due to limited immunogenicity, unfavorable tumor microenvironments with scarce immune infiltrates, and excessive accumulation of regulatory T cells [17]. Therefore, the potential to develop new therapeutic approaches that can convert immunologically ’cold’ or ’altered’ environments to ’hot’ environments has recently attracted increasing attention. [22,23].

**Figure 1:**
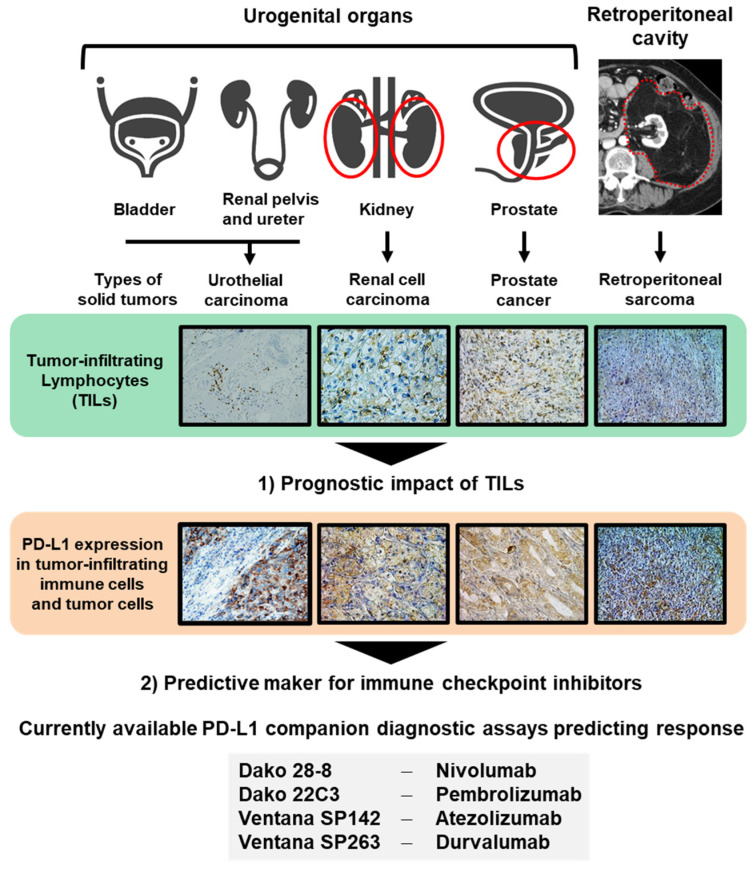
Two topics discussed in this review. Urologists should handle several malignancies arising from different organs, including the bladder, renal pelvis, ureter, kidney, prostate, and tissues of the retroperitoneal cavity. In this review, we discuss two topics: (**1**) the prognostic impact of tumor-infiltrating leukocytes (TILs) and (**2**) predictive markers for immune checkpoint inhibitors to shed light on lymphocyte migration in four solid tumors, the urothelial carcinoma, renal cell carcinoma, PCa, and retroperitoneal sarcoma. Currently available PD-L1 companion diagnostic assays predicting response to immune checkpoint inhibitors are shown.

As immune contexture can vary widely across types of tumor and tumor microenvironment, there exists a significant lack of clinically available definitive biomarkers that provide accurate predictions for treatment responses, especially in urogenital malignancies. Additionally, in this review, we will discuss the functional heterogeneity in the tumor-infiltrating immune system to explore its prognostic impact after surgery and other treatments. This review focuses on two main topics: (i) the prognostic impact of TILs and (ii) predictive biomarker for ICIs, to shed light on lymphocyte migration in four solid tumors which are urothelial carcinoma (UC), RCC, PCa, and retroperitoneal sarcoma (RSar) (Figure 1).

## 2. Methods

### 2.1. Literature Search

We searched for relevant papers published before 30 August 2020, by using the PubMed database with the following terms: “urothelial carcinoma” or “renal cell carcinoma” or “prostate cancer” or “sarcoma”, “tumor-infiltrating lymphocyte/TIL”, “tumor microenvironment”, “immune checkpoint inhibitor”, and “programmed death-ligand 1/PD-L1”. The inclusion criteria were as follows: studies which addressed the relevance between clinical outcomes including the treatment response and TILs, other immune cells, and PD-L1 positive cells. Any of immunohistochemical (IHC) staining analyses, hematoxylin and eosin (HE) staining analysis, and flow cytometry (FCM) analysis, and dataset analysis were allowed as methods for assessment of immune cells. Various types of retrospective studies and prospective clinical trials were included. If the same study was reported more than once, we selected the most recent publication with updated information.

### 2.2. Immunohistochemical Staining and Quantification in Tumor Tissues of UC Diseases Arising from Different Origins

IHC staining using paraffin-embedded, formalin-fixed tissue blocks was performed as previously described [16]. Bladder, renal pelvis, and ureter are three common primary origins of UC and compared in terms of the immune profile. The patients’ background is depicted in Table S1. The primary/secondary antibodies used in this study and the conditions are available in Table S2. A Histofine Simple Stain™ MAX PO (MULTI) kit (Nichirei Corporation, Tokyo, Japan) was used for peroxidase color development according to the manufacturer’s instructions. The staining results were evaluated by two investigators (Y.T. and Y.N.) who were blinded to any clinicopathological data. Data were expressed by box-and-whisker plots, in which the outliers are indicated as dots, and compared using the Kruskal-Wallis test, followed by the post hoc test (Dunn test). This observational study was approved by the ethics committee of the Nara Medical University, and informed consent from the participants was obtained in the form of written signature or opt-out on the web-site (reference ID: 1256).

## 3. Urothelial Carcinoma (UC)

### 3.1. Epidemiology and Current Issues of UC

UC is a histopathological type of cancer that typically arises from the urothelium of the bladder, renal pelvis, ureter, or urethral. UC of the bladder is the most common malignancy involving the urinary tract and is the sixth most common cancer in the United States [24]. Surgical resection is a standard treatment strategy for UC and other solid tumors. However, unresectable/metastatic UC is strongly associated with poor prognosis and requires multidisciplinary therapy, including chemotherapy, ICIs, and palliative radiotherapy. Wide use of pembrolizumab (KEYTRUDA^®^, Merck & Co. Inc., Kenilworth, NJ, USA) has clearly improved survival in selected patients with advanced UC [25]. As there exists a lack of prognostic markers after radical surgery and predictive markers for response to ICIs, we focused on the potential of TILs as a clinically available biomarker.

### 3.2. Clinical Impact of TILs in Patients with UC

Table 1 summarizes previous studies addressing the clinical relevance of TILs in patients with UC [26,27,28,29,30,31,32,33,34,35,36,37]. There are several types of treatment settings, including transurethral resection of bladder tumor (TURBT) with or without intravesical Bacillus Calmette-Guérin (BCG) for non-muscle invasive bladder cancer (NMBIC), radical cystectomy for muscle-invasive bladder cancer (MIBC), radical nephroureterectomy for upper urinary tract urothelial cancer (UTUC), and systemic chemotherapy for metastatic UC. Our group previously reported that a higher number of tumor-infiltrating regulatory T cells (Tregs) was associated with a higher risk of treatment failure [26]. This result was reasonable in terms of the hypothesis that pre-BCG baseline status of Th1/Th2 balance and Treg recruitment in the tumor microenvironment, or both, could influence the response to BCG [38]. Wahlin et al. demonstrated that CD8+ and FoxP3+ TILs in cystectomy specimens were independently associated with better outcomes, which disagrees with our previous data [26,32]. Additionally, this association was only observed in CD8+ TILs in TURBT specimens [32]. However, we understand that the data can be affected by the type of specimen and patient background, including treatment and race. Due to accessibility, most studies have utilized IHC staining analyses using archival paraffin-embedded tissues. Kawashima et al. evaluated nine extracellular surface markers measured by flow cytometry in freshly resected UC specimens [33]. In their study, Kawashima et al. classified tumors into the CD4 T-cell-dominant group and the immunologically activated group according to the immunologic condition, concluding that the latter group showed significantly poorer outcomes in patients with MIBC. Evaluation with freshly resected UC specimens is costly, time consuming, and requires effort; however, the results obtained are often robust and reliable. Overall, aside from the method of evaluation and interpretation, a consensus has not yet been reached regarding the prognostic value of TILs in patients with UC.

### 3.3. Predictive Biomarkers for Response to ICIs in UC

As of August 2020, five PD-1/programmed death-ligand 1 (PD-L1) inhibitors have been approved for the treatment of locally advanced or mUC. The U.S. Food and Drug Administration and the European Medicines Agency require IHC PD-L1 testing prior to first-line use with pembrolizumab and atezolizumab in platinum-ineligible patients [58]. Table 2 summarizes key clinical trials of ICIs for patients with UC refractory to chemotherapy [25,59,60,61,62,63]. There is a substantial proportion of patients who are less likely to benefit from ICIs, and the majority of the trials have so far explored the potential of PD-L1-related biomarkers to predict responses to ICIs in mUC. However, differences in antibodies, staining platforms, scoring algorithms and cut-off systems between trials have raised questions about interchangeability and comparability for diagnostic use, which could lead most pathologists to non-workable situation [58]. Used PD-L1 testing system in clinical trials are the Dako 28-8 for nivolumab, the Dako 22C3 for pembrolizumab, Ventana SP142 for atezolizumab, and the Ventana SP263 assays for durvalumab. Trials for pembrolizumab, atezolizumab, and durvalumab included combined assessments of PD-L1 staining of tumor cells and tumor-infiltrating immune cells or a single assessment of tumor-infiltrating immune cells, while the trials for nivolumab (CheckMate) included only PD-L1 expression in tumor cells [25,59,61,62,63]. CheckMate 032 did not check the expression of cytotoxic T-lymphocyte antigen 4 (CTLA-4) expression [60]. Expression of immune checkpoint proteins such as PD-1, PD-L1, and CTLA-4 can vary widely among primary and metastatic lesions and are affected by intratumor heterogeneity. A significant lack of correlation between the expression of these proteins and clinical outcomes/responses hampers the establishment of a Schottker-driven immunotherapy strategy.

### 3.4. The Immunological Profile in the Tumor Microenvironment of UC Arises from Different Primary Origins

**Figure 2:**
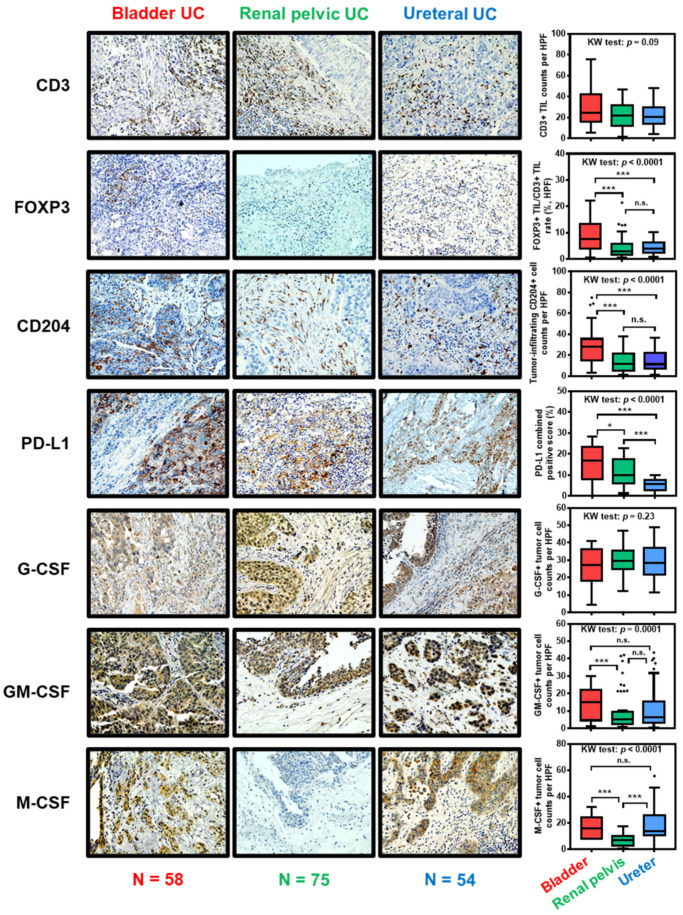
Difference in tumor immune microenvironments in urothelial carcinoma: comparison of primary tumor origins. The left panels are representative images of immunohistochemical staining of seven immune-related markers using surgically resected formalin-fixed paraffin-embedded specimens. Data were expressed by box-and-whisker plots, in which the outliers are indicated as dots, and compared using the Kruskal-Wallis (KW) test, followed by the post hoc test (Dunn test). * *p* < 0.05, *** *p* < 0.01, n.s., not significant. Abbreviations: UC, urothelial carcinoma; TIL, tumor-infiltrating leukocyte; HPF, high power field (magnification, ×400); PD-L1, programmed death-ligand 1; G-CSF, granulocyte colony-stimulating factor; GM-CSF, granulocyte-macrophage colony-stimulating factor; M-CSF, macrophage colony-stimulating factor.

Bladder, renal pelvis, and ureter are three common primary origins of UC. Although bladder UC, renal pelvic UC, and ureteral UC have many similarities, there are anatomical, biological, and molecular differences that should be considered as three distinct urothelium-derived malignancies [77]. Yates et al. reported significant differences in the genetic (microsatellite instability) and epigenetic (hypermethylation) backgrounds of bladder UC and UTUC [78]. Moreover, we have previously demonstrated that the subsequent NMIBC after radical nephroureterectomy for primary UTUC had a poorer prognosis after intravesical BCG compared to that in primary NMIBC [79]. This finding suggested that the primary origin is associated with an inherently poor response to BCG. However, all clinical trials of ICIs for metastatic UC clustered with UC diseases arising from different origins. Figure 2 shows representative images of IHC staining of seven immune-related markers using surgically resected UC specimens. Our previous works have presented the oncogenic or anti-tumoral effect of endogenous and exogenous colony-stimulating factors (CSFs) in UC [80,81,82]. In addition, accumulating evidence revealed that CD204, but not CD163, positive tumor-associated macrophages are associated with the aggressive behavior of various cancers including UC [16,83]. Thus, we included three CSFs and CD204 in the IHC analysis. The patients’ background and primary/secondary antibodies used in this study are depicted in Tables S1 and S2. Significant differences in the density of FOXP3-positive TILs, CD204-positive tumor-infiltrating macrophages, PD-L1-positive cells, granulocyte-macrophage colony-stimulating factor (GM-CSF), and macrophage colony-stimulating factor (M-CSF) were observed in the quantitative analysis. In the immunotherapy era, we may need to consider differences in the immunological profile among the disparate triplets.

## 4. Renal Cell Carcinoma (RCC)

### 4.1. Epidemiology and Current Issues of RCC

RCC accounts for approximately 2.2% of all cancers and is estimated to cause over 170,000 annual deaths globally [84]. RCC has been regarded as an immunogenic tumor, known as “hot tumor”, and is thought to weaken host immunity in order to enhance tumor growth. This feature has encouraged urologists to use immunotherapies including interleukin-2, interferon-alpha, and ICIs [81,82]. In contrast, approximately 30% of patients with RCC present with metastases, and recurrent disease develops in approximately 40% of patients previously treated for localized RCC [83]. Recently, the development of ICIs has significantly improved prognosis in advanced or metastatic RCC [64,65,66,84,85,86,87]. As there are still a large number of patients with advanced RCC who fail in these treatments, there is an urgent clinical need to identify predictive markers to improve treatment efficacy.

### 4.2. Clinical Impact of TILs in Patients with RCC

Table 1 summarizes previous studies addressing the clinical relevance of TILs in patients with RCC [39,40,41,42,43,44,45,46,47,48,49]. Most of these studies were intended for advanced or metastatic RCC and several types of treatment settings, including radical nephrectomy and systemic treatment with molecular targeted drugs. The clinical significance of TILs in patients with RCC has been reported over the past three decades.

In 1992, Igarashi et al. showed that CD8+/CD11- cells, which inhibit antibody production, increased and CD4+/CD45RA- cells, which introduced antibody-producing cells, decreased along with tumor progression [39]. Subsequent studies using IHC and flow cytometry analyses revealed that the complex interactions between tumor cells and TILs such as cytotoxic T cells, Tregs, exhausted T cells, and B cells were associated with the acceleration or suppression of tumor growth, thereby affecting the prognosis of RCC patients [40,41,42,43,44,45,46,47,48]. With regard to Tregs, migration of CD4+Foxp3+ T cells to the tumor microenvironment is an independent factor for poor prognosis [43,44,45]. However, Siddiqui et al. have shown that CD4+CD25+FoxP3+ T cells are not associated with prognosis, while an increase in CD4+CD25+FoxP3- T cells was significantly associated with poor prognosis [46]. Similar to Tregs, there are several subtypes of CD8+ TILs. An increase in the number of CD8+ TILs in the tumor microenvironment was associated with prolonged prognosis in RCC [41,43,49], while the increase in exhausted T cells, which belong to CD8+ T cells, was associated with poor prognosis in RCC [47,48]. There are various subtypes of CD4+ T cells and CD8+ T cells, and the difference in one cell-surface marker can be a switch for a completely opposite function in tumor progression. These complex mechanisms make it difficult to understand the various roles of each immune cell type. In contrast, ICIs use these complex mechanisms and enable the switch to escape from antitumor suppression. Overall, the clinical significance of TILs has not yet been fully elucidated. However, TILs may have the potential to improve the clinical outcome in patients treated with molecular targeted drugs and ICIs.

### 4.3. Predictive Biomarkers for Response to ICIs in RCC

As of August 2020, five PD-1/PD-L1/CTLA-4 inhibitors have been approved for the treatment of locally advanced or metastatic RCC. Table 2 summarizes key clinical trials (limited to phase II or III) of ICIs for advanced/metastatic RCC [64,65,66,67,68,69,70]. These clinical trials showed that ICIs were superior to conventional molecular targeted drugs such as tyrosine kinase inhibitors (TKIs) and mammalian target of rapamycin inhibitors (mTORis). Moreover, most of these clinical trials evaluated the efficacy of combination therapy with one ICI plus one targeted drug compared to that of TKI or mTORi, which was different from the one used in the trial arm. Combination therapies have shown a higher objective response rate (ORR). Careful interpretation of outcomes such as progression-free survival (PFS) and overall survival (OS) is needed. Additionally, most of these clinical trials have investigated the association between PD-L1 expression in tumor cells and outcomes. The CheckMate 025, KEYNOTE-426, JAVELIN Renal 101, CheckMate 214, BTCRC-GU14-003, and IMmotion151 trials investigated the association between PD-L1 expression in tumor cells and prognosis as a subgroup analysis, and these trials showed that high PD-L1 expression was associated with a treatment benefit for ICIs [64,66,67,68,69,70]. Interestingly, CheckMate 214 revealed that high expression of PD-L1 had a high rate of mortality in patients who received sunitinib [68]. This indicates the potential benefit of sequential treatment with ICIs and TKI/mTORis, which could create a favorable immune microenvironment. In contrast, there has been limited evidence for predictive biomarkers for ICI responses. One major limitation is that PD-L1 expression in tumor cells is the only immune checkpoint protein investigated in clinical trials. Thus, further research is necessary to establish a novel biomarker-driven immunotherapy strategy.

## 5. Prostate Cancer (PCa)

### 5.1. Epidemiology and Current Issues of PCa

PCa is the most common malignancy in men. The estimated lifetime risk of prostate cancer diagnosis is 13%, with a mortality to incidence ratio of 20% [88,89]. Prostatectomy and radiotherapy are standard curative treatments for localized PCa. Moreover, androgen deprivation therapy (ADT) is the standard primary therapy for metastatic PCa, because it is initially androgen-dependent and has a good response to ADT. However, some aggressive subsets progress to an androgen independent state, resulting in castration-resistant PCa (CRPC).

Various treatments, including androgen receptor-axis-targeted (ARAT) agents such as abiraterone [90] and enzalutamide [91], chemotherapy such as docetaxel [71] and cabazitaxel [72], a cellular vaccine called sipuleucel-T [92], and a radiopharmaceutical radium-223 [93] are available for patients with metastatic CRPC. Although the latest ICIs have shown strong antitumor activity in many tumors types, ICIs for advanced PCa and metastatic CRPC remain challenging [94,95,96,97].

### 5.2. Clinical Impact of TILs in Patients with PCa

As TILs are not abundant in the microenvironment of primary PCa [98,99] and PD-L1 expression in PCa cells is scarce [100], PCa is thought to be an immunologically “cold tumor”. This feature may limit the success of ICI trials for advanced PCa and metastatic CRPC. However, two studies demonstrated that sufficient TILs were detected in post-ADT tumors [50,101], suggesting that CRPC could be a potential target of ICIs. Sorrentino et al. demonstrated that ADT induced abundant T cell infiltration in both benign glands and tumor tissues in human prostates [101]. The authors also showed that an increased number of Foxp3+CD25+CD127- Tregs was detected after ADT [50]. Previous studies addressing the correlation between TILs and post-treatment survival in patients with PCa are listed in Table 1 [50,51,52,53]. A high CD8+/Foxp3+ ratio in prostate tissues treated with neoadjuvant ADT was identified as a good prognostic factor after prostatectomy [50]. On the other hand, Ness et al. demonstrated that high density of CD8+ lymphocyte infiltration, especially in the tumor epithelial area, was identified as an independent poor prognostic factor for biochemical-failure survival after radical prostatectomy [51], which differs from the data reported by Sorrentino et al. [50]. Nardone et al. revealed that increased expression of CD8+ and CCR7+ TILs is associated with longer PFS and OS, whereas increased PD-1 and Foxp3+ Treg expression was associated with longer PFS and OS in patients treated with salvage radiotherapy after radical prostatectomy [52]. Overall, there is a lack of sufficient evidence regarding the prognostic value of TILs in patients with prostate cancer.

### 5.3. Predictive Biomarkers for Response to ICIs in PCa

Unfortunately, there are no randomized control trials that demonstrated the benefit of ICIs for metastatic CRPC. Two studies analyzed biomarkers predicting the response to ICIs in patients with metastatic CRPC. The KEYNOTE-199 phase II study analyzed the response to pembrolizumab for 258 patients with metastatic CRPC who had previously treated with docetaxel or ARATs [71]. PSA response rates were 6% in cohort 1 (PD-L1-positive group), 8% in cohort 2 (PD-L1-negative group), and 2% in cohort 3 (bone-predominant disease, regardless of PD-L1 expression), respectively. Median OS were 9.5 months in cohort 1, 7.9 months in cohort 2, and 14.1 months in cohort 3, respectively. Moreover, this study analyzed correlation between the status of the homologous recombination repair genes and the response to pembrolizumab. Response duration was 4.4 months in patients with an ATM mutation and more than 21.8 months in patients with a BRCA2 mutation. However, it is difficult to draw a conclusion regarding predictive biomarkers of response to ICIs because of the low number of responders in this study. Another single-arm phase II trial analyzed the efficacy of pembrolizumab plus enzalutamide for 28 patients with metastatic CRPC [53]. Five (18%) of 28 patients had PSA decline of 50 % or greater. For entire cohort, the median OS was 22 months. The median OS for 5 responders was 41.7 months. Thirteen-patients could be identified tumors by base line biopsy for metastatic lesions. In one responder and two non-responders, PD-L1 was detected on TILs. The frequency of granzyme B+ CD8+ T cell and perforin+ CD8+ T cell was higher in responder than in non-responder, suggesting that the clinical benefit of pembrolizumab plus enzalutamide might require the pre-exiting tumor-reactive CD8+ T cell. The frequency of PD-L1 expression in patients with metastatic CRPC was 31.6 % in previously publication [102], which was higher than in this study. Based on these findings, there are a significant lack on evidence regarding correlation between TILs (i.e., PD-1 and PD-L1) and outcome/response to ICIs in patients with metastatic CRPC because of the nature of prostate cancer, considering scarce TILs and low PD-L1 expressions. Therefore, further studies were warranted to demonstrate the anti-tumor activity of immunotherapy including not only PD-1 inhibitor monotherapy but also combination-therapy with CTLA-4 inhibitor.

## 6. Retroperitoneal Sarcoma (RSar)

### 6.1. Epidemiology and Current Issues of RSar

Soft tissue sarcomas (STSs) are rare tumors with the incidence less than 1% of all adult solid malignancies [103]. STSs can occur in any anatomic region and those arising from the retroperitoneal cavity are classified “RSar” and a urological malignancy, accounting for only 12–15% of all STSs [104]. Extended surgical resection of primary tumor and the surrounding vital organs is the mainstay of the treatment of RSar [105]. Although perioperative radiotherapy and chemotherapy are available to improve the rate of complete resection, it is complicated to define the optimal treatment strategies due to the disease heterogeneity and variety of tumor subtype.

Recurrent, unresectable, and metastatic RSars are indicated for systemic therapy. Chemotherapeutic drugs such as doxorubicin plus dacarbazine, doxorubicin plus ifosfamide, or doxorubicin alone has been administered as the first-line treatment for advanced sarcomas [106]. Recently, accumulating evidences have demonstrated the potential benefit of immunotherapies, especially ICIs (Table 2) [73,74,75,76,107,108]. Because the response to chemotherapy and immunotherapy is not expected in all the patients and the predictive markers are not available, there are still many limitations in the clinical management of advanced RSar. Here, we summarize the potential of TILs and PD-L1 expression as a clinically available biomarker for RSar.

### 6.2. Clinical Impact of TILs in Patients with RSar

We previously investigated the clinical significance and prognostic implications of intratumoral PD-L1, PD-L2, PD-1, and Ki-67 expression in patients with RSar [54]. Among these markers, only high expression of PD-1 in the TILs was a possible predictor of postoperative recurrence. Interestingly, observation of several clinicopathological parameters showed that high levels of serum lactate dehydrogenase (LDH) were significantly correlated with high intratumoral expressions of PD-L1 and PD-L2 [54]. This novel finding implies that elevated levels of serum LDH might be associated with response to the treatment of PD-1/PD-L1 blockade. Tseng et al. analyzed the intratumoral adaptive immune response in well differentiated- and dedifferentiated-retroperitoneal liposarcomas using isolation of TILs from surgically resected tumors followed by flow cytometry [74,75]. Although the majority of TILs were CD4+ T cells, cytotoxic CD8+ T cells accounted for 20% of CD3+ T cells. Notably, 65% of CD8+ T cells were positive for the PD-1. Immune cell aggregates evaluated by IHC was associated with worse prognosis in both well differentiated and dedifferentiated retroperitoneal liposarcoma, suggesting that an adaptive immune response was present in the liposarcomas but may be hindered by Immune cell aggregates among other possible microenvironmental factors [56]. Moreover, Yan et al. investigated the density of TILs in various types of retroperitoneal liposarcomas [57]. The proportion of TILs was the highest in the dedifferentiated retroperitoneal liposarcoma and the lowest in pleomorphic liposarcoma. The authors demonstrated that patients with higher FOXP3+ Treg or PD-1/PD-L1+ cells tended to be a poor prognostic factor. Heterogeneous TILs distribution was found in 50% patients and tended to correlate with favorable disease-free-survival [56]. In spite of the accumulating evidence, the clinical impact of TILs in RSar still remains uncertain, especially in other types of RSar such as leiomyosarcoma and undifferentiated pleomorphic sarcoma, formally known as malignant fibrous histiocytoma.

### 6.3. Predictive Biomarkers for Response to ICIs in RSar

Because RSar is an extremely rare tumor, no clinical trial for the treatment with ICIs has evaluated the clinical benefit only in advanced RSar. Majority of clinical trials included the STS and bone sarcoma (BS). The SARC028 phase II study was the first clinical trial evaluating ICIs for advanced sarcomas [73], in which 40 patients in each disease cohort were treated pembrolizumab. A tumor was considered positive for PD-L1 expression if more than 1% of its cells showed membranous staining. PD-L1 expression was observed in only 5% of patients; both were undifferentiated pleomorphic sarcoma and responded to pembrolizumab treatment. Another combination therapy with talimogene laherparepvec (T-VEC) plus pembrolizumab for locally advanced or metastatic sarcomas has shown promising results [75]. T-VEC is a biopharmaceutical drug and shows anti-tumor effect by increase in tumor-specific immune activation via augmenting antigen presentation and T-cell priming. Of all patients showing the treatment response, pre-treatment tumor sample had aggregates of CD3+/CD8+ TILs at the infiltrating tumor edge. However, only 1 patient demonstrated PD-L1 positive at baseline; this patient achieved a partial response. In contrast, 64% of the posttreatment tumor were PD-L1 positive and 55% of patients converted from PD-L1 negative to positive after treatment [75]. The phase II study of the combination of axitinib plus pembrolizumab for advanced or metastatic sarcomas was published in 2019 [76]. PD-L1 expression was positive in 52% of patients with evaluable tumor biopsy samples. However, neither PD-L1 positivity nor increased tumour-infiltrating lymphocyte score correlated with progression-free survival of longer than 6 months or achieving a partial response. Currently, there is no predictive biomarker available for the treatment of ICIs in advanced sarcomas including RSar.

## 7. Limitations and Current Perspective Regarding the Assessment of TILs

We focused exclusively on the potential of TILs as a prognostic or predictive marker. Relevant studies have been performed in a retrospective manner and in relatively small cohorts. The definitions of TILs, such as inclusion of intratumoral TILs and/or stromal TILs, and the scoring methodology varied among studies. These inconsistencies hinder comparisons across studies and extrapolation of findings to clinical practice. Large studies investigating the potential prognostic value of TILs as assessed on HE staining are lacking. International Immuno-Oncology Biomarker Working Group on Breast Cancer has developed the international guidelines regarding the assessement of TILs on HE-stained slides without any specific staining [109]. The purpose of this group is to develop standards on the assessment of immuno-oncology biomarkers to aid pathologists, clinicians and researchers in their research and daily clinical practice. International Guidelines on TIL-assessment in solid tumors Part 2 provided the recommendation in assessment of genitourinary carcinomas. According to the guidelines [109], separate reporting of intratumoral TILs and stromal TILs is recommended—this is based on the context of atezolizumab treatment in mUC, where the PD-L1 “immune cell” score is derived from the stromal TILs score [110]. In addition, special care should be taken to avoid areas of tumor zones with necrosis, coagulation artifact, and previous biopsy sites, which is a common finding in resected specimens of bladder tumor. However, detailed tutorial for RCC, PCa, and sarcoma is not available because of insufficient data to make specific recommendations.

Early data showed the presence of TILs in UC was associated with a favorable prognosis [111]. As to early data of RCC, increased TILs, both CD4-positive and CD8-positive T cells, appear to related with high risk of post-nephrectomy recurrence and poor prognosis [112,113,114,115,116]. However, accumulating evidence, largely based on IHC quantification of different TIL subsets, have somehow turned conflicting results on the prognostic relevance of TILs (Table 1). Majority of reports on TILs in PCa have focused on the prognostic value of TILs, while few studies investigating the potential to predicting response to drug therapies. Most reports have shown the evidence for a relationship between the high TILs and increased risk of recurrence [117,118,119], metastasis [120], and poor cancer specific survival [121]. The result on the composition of TILs in are heterogeneous and sometimes conflicting, and the relationship between TILs and survival is still unclear in PCa [109]. Overall, we emphasis on the importance of uniform assessment of TILs and uniform comparison of study results in research practices.

## 8. Conclusions

As, an “immunotherapy tsunami”, in particular ICIs, has overtaken the oncological field in this decade, it is mandatory for Physicians to deepen knowledge about cancer immunity and tumor immune microenvironment. This review highlights comprehensively the following two topics: (i) prognostic impact of TILs and (ii) predictive maker for ICIs in four urological solid tumors: UC, RCC, PCa, and Sar. Although there is accumulating evidence that the density of TILs can serve as a prognostic biomarker and/or predictive biomarker for immunotherapies, inconsistency of TIL evaluation and interpretation for the results seems to hinder its clinical application. Unfortunately, across different solid malignancies, the response rate and predictive markers for ICIs may vary significantly. Multiple biomarkers including tumor-infiltrating immune cells, PD-L1 expression, other immune checkpoint protein expression, mRNA gene expression analysis, mismatch-repair deficiency, and tumor mutational burden may need to overcome disease heterogeneity and complex tumor immunity. Both identification of positive or negative predictive biomarkers of ICIs and development promising combination are required urgently to refine the clinical management of advanced urological malignancies. Further studies with large-scale cohorts and long follow-up periods to prove the clinical impact of novel prognostic/predictive biomarkers, followed by their adoption in clinical practice.

## Figures and Tables

**Table 1:** Studies for the clinical relevance of tumor-infiltrating lymphocytes (TILs) in urological malignancies and retroperitoneal sarcoma.

Types of Tumor	No. of Patients	Treatment	Makers or Assessment	Assay	Clinical Relevance	Reference No.
Urothelial Carcinoma (UC)						
NMIBC	154	TURBT followed by intravesical BCG	FOXP3, CD204	IHC	High Tregs and tumor-associated macrophages were associated with a high risk of intravesical recurrence.	[26]
NMIBC	115	TURBT	CD3, CD4, CD8, CD20, CD56, CD68, granzyme B	IHC	Low CD3+ TILs and CD8+ TILs were associated with a high risk of intravesical recurrence.	[27]
NMIBC	131	TURBT	CD4	IHC	High CD4+ TILs were associated with poor OS.	[28]
NMIBC	102	TURBT	CD8, CD66b	IHC	High tumor-infiltrating neutrophils and NLR were associated with poor OS. High TILs were related to longer OS.	[29]
MIBC	67	Radical cystectomy	CD3, CD8	IHC	High CD8+ TILs and CD3+ TILs in the invasion margin were associated with better DFS and OS.	[30]
MIBC	406	Radical cystectomy	CD3D, CD4, CD8A	mRNA (TCGA dataset)	High CD3D/CD4 ratio was associated with improved survival. The power was stronger in basal-squamous tumors.	[31]
MIBC	145	Radical cystectomy	CD8, FOXP3, CD20, PD-1, PD-L1	IHC	High density of CD8, FOXP3, CD20, and PD-1 was associated with a low risk of recurrence.	[32]
Bladder cancer and UTUC	52 and 18	Surgical resection	Nine extracellular surface markers	FCM	The immunologically activated group showed poorer PFS and CSS compared that in to the CD4+ T-cell-dominant group in bladder cancer. However, there was no significant difference in UTUC.	[33]
UTUC	162	Radical nephroureterectomy	PD-L1	IHC	High PD-L1 expression in tumor cells was associated with shorter CSS. High PD-L1 expression on TILs was associated with longer CSS.	[34]
UTUC	423	Radical nephroureterectomy	PD-1, PD-L1	IHC	High PD-1 level was associated with poor CSS and OS. In patients with organ-confined disease (pT2≤, N0/xM0), high PD-L1 was associated with a high risk of recurrence and poor OS.	[35]
UTUC	88	Radical nephroureterectomy	CD4, CD8, CD20, APE1, NTH1, OGG1, XRCC1, polβ, STING, IRF3, PD-L1, PD-L2	IHC	High CD8+ TILs were associated with poor DFS.	[36]
Metastatic UC	259	Platinum-based chemotherapy	Recommendations by an International TILs Working Group 2014	Hematoxylin and eosin staining	High TIL levels were associated with better OS after chemotherapy both in bladder cancer and UTUC.	[37]
Renal cell carcinoma (RCC)						
ccRCC	43	Untreated stage III/IV disease	CD4, CD45RA, CD8, CD11, HLA-DR, CD3, CD16, CD57	FCM	An increase in CD8+/CD11- and a decrease in CD4+/CD45RA- cells were observed along with the aggravation of tumor stage and grade.	[39]
ccRCC	473	Previously treated	Th17, CTL, Tregs, Th2	mRNA (TCGA dataset)	Long-lived patients have high levels of Th17 and CD8+ T cells, while short-lived patients have high levels of Tregs and Th2.	[40]
RCC	891	Untreated	M1 macrophages, M2 macrophages, memory CD4+ T, γδ T, CD8+ T, Tregs, naïve CD4+ T, NK cell, mast cells, B cells, DC, monocytes, plasma cells, neutrophils, eosinophils	CIBERSORT	CD8+ T cells were associated with prolonged OS. A higher proportion of regulatory T cells was associated with a poorer outcome. M1 macrophages were associated with a favorable outcome, while M2 macrophages indicated a poorer outcome.	[41]
Metastatic ccRCC	167	Previously treated	CD8, PD-1, TIM-3, LAG-3	IHC	A high percentage of CD8+/PD-1+/TIM-3-/LAG-3- cells correlated with high levels of T-cell activation and were associated with longer median irPFS and higher irORR.	[42]
ccRCC	199	Previously treated	PD-1, FOXP3	IHC	PD1-positive or FOXP3-positive lymphocytes can be used as significant prognostic indicators, and PD1 positivity could be very helpful in the prediction of latent distant metastasis.	[43]
Metastatic ccRCC	58	interleukin-2-based immunotherapy	FOXP3	IHC	Intratumoral FOXP3-positive regulatory immune cells significantly increased during interleukin-2–based immunotherapy, and high numbers of on-treatment FOXP3-positive cells were correlated with poor prognosis.	[44]
ccRCC	125	Radical nephrectomy or nephron-sparing surgery	CD4, FOXP3	IHC	Increased peritumoral Tregs are associated with a poorer prognosis.	[45]
ccRCC	170	Radical nephrectomy or nephron-sparing surgery	CD4, CD25, FOXP3	IHC	Increased number of CD4+CD25+Foxp3+ T cells was not associated with RCC death. In contrast, CD4+CD25+Foxp3- T cells, which may represent a unique set of Tregs or activated helper T cells, were significantly associated with the outcome.	[46]
RCC	97	Previously treated	CD45, CD3, CD4, CD8, CD45RA, ICOS, Tim3, CD25, PD-1, FOXP3	FCM	Tumor grade significantly correlated with dysfunction of both CD4+ and CD8+ TILs and the efficacy of nivolumab treatment.	[47]
Localized ccRCC	40	Radical nephrectomy or nephron-sparing surgery	CD3, CD4, CD8, CD45RA, CCR7, CD69, CD38, CD40L, ICOS, GITR, PD-1, TIM-3, CTLA-4, LAG-3, CD127, CD25	FCM	Infiltration with CD8+PD-1+Tim-3+Lag-3+ exhausted TILs and ICOS+ Tregs identified patients with deleterious prognosis who could benefit from adjuvant therapy with TME-modulating agents and checkpoint blockade.	[48]
Metastatic RCC	231	Tyrosine kinase inhibitors	CD8, PD-1, PD-L1	IHC	Increased numbers of CD8+ T cells are significantly associated with improved survival in patients with mRCC treated with TKIs. PD-1 could be used as a predictive and prognostic factor.	[49]
Prostate cancer (PCa)						
Localized PCa	126	Radical prostatectomy	CD8, FOXP3	IHC	High CD8+ TILs were significantly associated with good DFS, whereas FOXP3+Treg tumor infiltration was significantly correlated with poor DFS.	[50]
Localized PCa	535	Radical prostatectomy	CD8	IHC	A high density of CD8+ TILs is an independent negative prognostic factor for biochemical failure-free survival.	[51]
Biochemical recurence after radical prostatectomy	22	Salvage radiotherapy	PD-1, FOXP3	IHC	High PD-1 and FOXP3+ Treg tumor infiltration was significantly associated with short PFS.	[52]
Localized PCa	75	Radical prostatectomy	CCR4	IHC	CCR4+ Tregs are highly infiltrated in the prostate tissue with poor prognosis, with a strong potential to progress to CRPC.	[53]
Retroperitoneal sarcoma (RSar)						
RSar (various types)	51	Surgical resection	PD-1, PD-L1, PD-L2, Ki-67	IHC	The prognostic value of PD-L1, PD-L2, and PD-1 expression was evaluated, and only high expression of PD-1 was a possible predictor of postoperative recurrence.	[54]
RSar (WDLPS)	6	Surgical resection	CD4, CD8, CD20	IHC	CD8+ T cells were mostly seen in scattered gout of the tumor. CD4+ T cells were observed in clusters and follicles. CD20+ cells (B cells) were found almost exclusively in cluster and forming immature follicles.	[55]
RSar (WDLPS/DDLPS)	8	Surgical resection	CD3, CD4,CD8, PD-1, 4-1BB	IHC FCM	Cytotoxic CD8+ T cells accounted for 20% of CD3+ T cells. Notably, 65% of CD8+ T cells were positive for PD-1. Immune cell aggregates evaluated by IHC were associated with poorer prognosis in both well-differentiated and dedifferentiated retroperitoneal liposarcoma.	[56]
RSar (WDLPS/DDLPS/MLPS/PLPS)	56	Surgical resection	CD4, CD8, FOXP3, CD20, PD-1, PD-L1	IHC	Higher FOXP3+ Treg or PD-1/PD-L1+ cells tended to be associated with poor prognosis. Heterogeneous TIL distribution was found in 50% of patients and tended to correlate with favorable disease-free survival.	[57]

UC, urothelial carcinoma; NMIBC, non-muscle invasive bladder cancer; MIBC, muscle invasive bladder cancer; TURBT, transurethral resection of bladder tumor; IHC, immunohistochemical staining; TIL, tumor-infltrating lymphocyte; FCM, flow cytometry; Treg, regulatory T cell; UTUC, upper urinary tract urothelial cancer; NLR, neutrophil-to-leukocyte ration; CSS, cancer-specific survival; OS, overall survival; DFS, disease-specific survival; PFS, progression-free survival; NA, not available; ccRCC, clear cell type RCC; CRPC, castration resistant prostate cancer; WDLPS, well differentiated liposarcoma; DDLPS, dedifferentiated liposarcoma; MLPS, myxoid/round cell liposarcoma; PLPS, pleomorphic liposarcoma; PD-L1, programmed cell death ligand-1; PD-L2, programmed cell death ligand-2.

**Table 2:** Key trials of immune checkpoint inhibitors and association between treatment response and maker assessment in advanced urogenital malignancies.

Types of Tumor	No. of Patients	Treatment (Phase)	Outcomes and Response			Assay	Marker Assessment	Clinical Relevance of Maker Assessment	Reference No.
			OS	PFS	ORR				
Urothelial carcinoma (UC)									
Advanced Muc (JAVELIN Solid Tumor)	249	Second-line avelumab (Phase I)	6.5 months	1.7 months	17%	NA	NA	NA	[59]
Advanced mUC (CheckMate 032)	274	Platinum-pretreated nivolumab ± ipilimumab (Phase I/II)	10.4 months in NIVO3 7.4 months in NIVO3+IPI1 27.6 months in NIVO1+IPI3	2.8 months in NIVO3 2.6 months in NIVO3+IPI1 4.9 months in NIVO1+IPI3	26% in NIVO3 27% in NIVO3+IPI1 38% in NIVO1+IPI3	IHC Dako 28-8	PD-L1 expression in tumor cells	PD-L1 expression was not associated with ORR. High PD-L1 expression was associated with longer mOS.	[60]
Advanced mUC	191	Durvalumab (Phase I/II)	18.2 months	1.5 months	18%	IHC Ventana SP263	PD-L1 (combined assessment of PD-L1 staining of tumor cells and immune cells)	Tumor response to durvalumab was not associated with PD-L1 staining.	[61]
Advanced mUC (CheckMate 275)	270	Second-line Nivolumab (Phase II)	8.7 months	2.0 months	20%	IHC Dako 28-8	PD-L1 expression in tumor cells	OR was observed in 28% of patients with PD-L1 expression of 5% or greater, 24% of patients with PD-L1 expression of 1% or greater, and 16% of patients with PD-L1 expression of less than 1%.	[62]
Advanced mUC (KEYNOTE-045)	542	Second-line Pembrolizuma vs. chemotherapy (Phase III)	10.3 vs. 7.4 months HR: 0.73 *p* value: 0.002	2.1 vs. 3.3 months HR: 0.98 *p* value: 0.42	21% vs. 11%	IHC Dako 22C3	PD-L1 combined positive score (CPS; the percentage of PD-L1-expressing tumor and infiltrating immune cells relative to the total number of tumor cells)	Treatment response was similar in patients with a CPS of 10% or more.	[25]
Advanced mUC (IMvigor211)	931	Second-line Atezolizumab vs. chemotherapy (Phase III)	11.1 vs. 10.6 months HR:0.87 *p* value: 0.41	2.4 vs. 4.2 months HR: 1.01	23% vs. 22%	IHC Ventana SP142	PD-L1 expression on <1% [IC0], 1% to <5% [IC1], and ³5% of tumor-infiltrating immune cells [IC2/3]	Atezolizumab was not associated with longer OS than chemotherapy in patients with IC2/3.	[63]
Renal cell carcinoma (RCC)									
Metastatic RCC (BTCRC-GU14-003)	61	Second or Third-line pembrolizumab plus bevacizumab (Phase Ib/II)	NA at the median follow-up of 28.3 months	20.7 months	60.90%	IHC Dako 22C3	PD-L1 expression in tumor cells	Patients with tumors overexpressing PD-L1 > 0 showed a trend toward better PFS after 20 months, but there was no statistical difference in overall PFS.	[64]
Metastatic RCC	30	Second or Third-line lenvatinib plus pembrolizumab (Phase Ib/II)	NA	19.8 months	70%	NA	NA	NA	[65]
Advanced RCC (KEYNOTE-426)	861	First-line pembrolizumab plus axitinib vs. sunitinib (Phase III)	HR: 0.53 *p* value: <0.0001	15.1 vs. 11.1 months HR = 0.69 *p* value: <0.001	59.7% vs. 35.7%	IHCDako 22C3	PD-L1 combined positive score (the percentage of PD-L1+ tumor and infiltrating immune cells/the total tumor cells)	The benefit of pembrolizumab plus axitinib was observed in patients with tumors expressing PD-L1 expression and those with tumors without PD-L1 expression.	[66]
Advanced RCC (JAVELIN Renal 101)	886	First-line avelumab plus axitinib vs. sunitinib (Phase III)	12.0 and 11.5 months HR: 0.78 *p* value: 0.14	13.8 vs. 8.4 months HR = 0.69 *p* value: <0.001	51.4% vs. 25.7%	IHC Ventana SP263	PD-L1 expression in tumor cells	ORR among patients with PD-L1–positive tumors who received avelumab plus axitinib was twice as that in patients who received sunitinib (55.2% vs. 25.5%, respectively).	[67]
Advanced or Metastatic RCC (CheckMate 214)	1096	First-line nivolumab plus ipilimumab vs. sunitinib (Phase III)	NR and 26.6 months HR: 0.66 *p* value: <0.0001	8.2 vs. 8.3 months HR = 0.77 *p* value: 0.0014	42% vs. 29%	IHC Dako 28-8	PD-L1 expression in tumor cells	Partial responders and complete responders to nivolumab plus ipilimumab both had higher baseline tumor PD-L1 expression than that in non-responders.	[68]
Advanced or Metastatic RCC (IMmotion151 trial)	915	First-line atezolizumab plus bevacizumab vs. sunitinib (Phase III)	33.6 and 34.9 months HR = 0.93 *p* value: 0.48	11.2 and 8.4 months HR = 0.83 *p* value: 0.022	37% vs. 33%	IHC Ventana SP142	PD-L1 expression in tumor cells	In the PD-L1 positive population, the median progression-free survival in the atezolizumab plus bevacizumab group was significantly longer than that in the sunitinib group. PD-L1 can be used as a supporting tool for treatment selection.	[69]
Advanced or Metastatic RCC (CheckMate 025)	821	Second or Third-line Nivolumab vs. everolimus (Phase III)	25.0 and 19.6 months HR: 0.73 *p* value: 0.002	4.6 vs. 4.4 months HR = 0.88 *p* value: 0.11	25% vs. 5%	IHC Dako 28-8	PD-L1 expression in tumor cells	Higher levels of PD-L1 expression are associated with poorer survival, while it does not support PD-L1 as a marker of treatment benefit.	[70]
Prostate cancer (PCa)/castration resistant PCa (CRPC)									
Metastatic CRPCKEYNOTE-199	258	Pembrolizumab after docetaxel or ARATs	9.5, 7.9, and 14.1 months in cohort 1 (PD-L1 positive), cohort 2 (PD-L1 negative), and cohort 3 (Bone-predominant)	2.1, 2.1, and 3.7 months in cohort 1 (PD-L1 positive), cohort 2 (PD-L1 negative), and cohort 3 (Bone-predominant)	7% and 2 % in cohort 1 (PD-L1 positive) and cohort 2 (PD-L1 negative)	IHC Dako 22C3	PD-L1 expression and aberrations of homologous recombination repair (HRR) gene in tumor cells	There were no significant differences in the response to pemblolizumab between the PD-L1-positive and -negative groups.	[71]
Metastatic CRPC	28	Pemblolizumab and enzalutamide	22.2 months	3.7 months (PSA-PFS)	18%	IHC and FCM	PD-L1 expression in tumor cells	The frequency of granzyme B+ CD8+ and perforin+CD8+ T cells were higher in responders those that in non-responders.	[72]
Retroperitoneal sarcoma (RSar)									
STS and BS (SARC028)	40 and 40	Pembrolizumab (phase II)	12.3 months (95% CI, 8.5–18.3)	4.2mounths (95% CI, 2.0–5.3)	NA	IHC Dako 22C3	Score was expressed as percentage of tumour cells positive for PD-L1. A tumour was considered positive for PD-L1 expression if more than 1% of its cells showed membranous staining.	PD-L1 expression was observed in only 5% of samples; both were UPS and responded to therapy. Pembrolizumab showed encouraging activity in patients with UPS or DDLPS.	[73]
Advanced or metastaic STS and BS (Alliance A091401)	43 and 42	Nivolumab vs. Nivolumab+ipilimmab (phase II)	10.7 and 14.3 months (95% CI, 5.5–15.4 and NA)	1.7 and 4.1 months (95% CI, 1.4–4.3 and 1.4–4.7)	NA	NA	NA	Treatment with nivolumab plus ipilimumab in an unselected cohort of heavily treated patients with advanced sarcoma, achieved a proportion of 16% of 38 patients with confirmed objective responses, which is similar to the results obtained with standard chemotherapy.	[74]
Locally advanced or metastaic sarcoma	20	T-VEC plus pemblolizumab (phase II)	18.7 months (95% CI, 12.3–NA)	4.3 months (95% CI, 3.2–NA)	30%	IHC	PD-L1 tumor membrane expression and CD3+/CD8+ TILs at the infiltrating edge of the tumor. The patients underwent pretreatment and posttreatment tumor biopsies.	The data show that 64% of the posttreatment tumors were PD-L1 positive and 55% of patients converted from PD-L1 negative to PD-L1 positive after treatment.	[75]
Advanced sarcomas including alveolar soft-part sarcoma	33	Axitinib plus pembrolizumab (phase II)	18.7 months (95% CI, 12.0–NA)	4.7 months (95% CI, 3.0–9.4)	25%	IHC	PD-L1 expression in sarcoma cells	PD-L1 expression was positive in 52% of patients with evaluable tumor biopsy samples. Neither PD-L1 positivity nor increased TIL score correlated with progression-free survival of longer than 6 months or with achieving a partial response.	[76]

OS, overall survival; PFS, progression-free survival; ORR, objective response rate; UC, urothelial carcinoma; HR, hazard risk; IHC, immunohistochemical staining; PD-L1, programmed cell death ligand-1; CPS, combined positive score; RCC, renal cell carcinoma; NR, not reached; RSar, retroperitoneal sarcoma; STS, soft-tissue sarcoma; BS, bone sarcoma; CI, confidence interval; NA, not available; FCM, flow cytometry; Treg, regulatory T cell; NLR, neutrophil-to-leukocyte ration; NA, not available; TIL, tumor-infiltrating leukocytes.

## Supplementary Materials

The following are available online at https://www.mdpi.com/2072-6694/12/11/3153/s1, Table S1: The patients’ background used for immunohistochemical staining analysis, Table S2: Primary and secondary antibodies used for immunohistochemical staining analysis.
