# Supplementary material for: Clinical Impact of Tumor-Infiltrating Lymphocytes and PD-L1-Positive Cells as Prognostic and Predictive Biomarkers in Urological Malignancies and Retroperitoneal Sarcoma
Source: Cancers (Basel). 2020 Oct 27;12(11):3153. doi: 10.3390/cancers12113153 (PMC7692684; doi:10.3390/cancers12113153)
Supplement: Supplementary file 1 [file cancers-12-03153-s001.pdf]

Supplementary Materials:

Clinical Impact of Tumor-Infiltrating Lymphocytes and PD-L1-Positive Cells as Prognostic and Predictive Biomarkers in Urological Malignancies and Retroperitoneal Sarcoma

Makito Miyake [1,*], Shunta Hori [1], Takuya Owari [1], Yuki Oda [1], Yoshihiro Tatsumi [1], Yasushi Nakai [1], Tomomi Fujii [2] and Kiyohide Fujimoto [1]

Table 1. The patients' background used for immunohistochemical staining analysis.

Primary Tumor Origin of Urothelial Carcinoma		Bladder	Renal Pelvic	Ureter
Total		58	75	54
Age, years-old (median, range)		72 (52-86)	67 (46-86)	70 (48-88)
Pathological T categoly (TNM Classification of Malignant Tumours, 8th ed.)	T1	-	30 (40%)	23 (43%)
	T2	29 (50%)	10 (13%)	10 (19%)
	T3	17 (29%)	28 (37%)	17 (32%)
	T4	12 (21%)	7 (9%)	4 (7%)

Table 2. Primary and secondary antibodies used for immunohistochemical staining analysis.

Antigen	Provider	Catalogue Number	Clone	Clonality	Antigen Retrieval	Dilution Rate	Incubation Time	Incuvation Temperature
CD3	Santa Cruz	sc-20047	PC3/188A	Mouse monoclonal	10mM Citric Acid, pH 6.0 Autoclave for 20 min	1:50	Over night	4 °C
FOXP3	Abcam	ab20034	236A/E7	Mouse monoclonal	10mM Citric Acid, pH 6.0 Autoclave for 20 min	1:100	Over night	4 °C
CD204	TransGenic	KT022	SRA-E5	Mouse monoclonal	10mM Citric Acid, pH 6.0 Autoclave for 20 min	1:2,000	Over night	4 °C
PD-L1	Cell Signaling Technology	13684	E1L3N	Rabbit monoclonal	10mM Citric Acid, pH 6.0 Autoclave for 20 min	1:100	Over night	4 °C
G-CSF	IBL	#11041	4-12-2	Mouse monoclonal	10mM Citric Acid, pH 6.0 Autoclave for 20 min	1:50	Over night	4 °C
GM-CSF	Origene	PP1101P1	NA	Rabbit polyclonal	10mM Citric Acid, pH 6.0 Autoclave for 20 min	1:400	Over night	4 °C
M-CSF	Abcam	ab52864	EP1179Y	Rabbit polyclonal	10mM Citric Acid, pH 6.0 Autoclave for 20 min	1:500	Over night	4 °C
Anti-mouse IgG and anti-rabbit IgG biotin-conjugated secondary antibodies	Nichirei Bioscience	426072	-	Goat polyclonal	-	ready-to-use	10 min	Room temparature

PD-L1, program death ligand-1; G-CSF, granulocyte colony-stimulating facto; GM-CSF, granulocyte-macrophage colony-stimulating factor; M-CSF, macrophage colony-stimulating factor.; IgG, immunoglobulin G; NA, not available
